# Preparation and Paclitaxel-Loading of Regenerated Chitin Nanoparticles

**DOI:** 10.3390/polym17223036

**Published:** 2025-11-16

**Authors:** Yanping Li, Dan Qiu, Xuechen Zhuang, Zhiduo Pei, Tao Li, Shanggui Deng

**Affiliations:** 1 School of Food and Pharmacy, Zhejiang Ocean University, Zhoushan 316022, China; li12031130@163.com (Y.L.); dengshanggui@163.com (S.D.); 2 School of Biological and Chemical Engineering, NingboTech University, Ningbo 315199, China; p2822485320@163.com (Z.P.); 15168197989@163.com (T.L.); 3 School of Materials and Chemical Engineering, Ningbo University of Technology, Ningbo 315211, China

**Keywords:** chitin, chitin nanoparticles (ChNps), chitin nanoparticles-paclitaxel (ChNps–PTX), regeneration, nano-strip PTX, crystallinity

## Abstract

This study aims to investigate biodegradable and biocompatible chitin as a natural carrier for loading the hydrophobic anti-cancer drug paclitaxel (PTX) for the first time, resulting in a new loading system of chitin nanoparticles–paclitaxel (ChNps–PTX). The well-dispersed chitin nanoparticles (ChNps) and ChNps–PTX were prepared via a water-dripping regeneration method. The functional groups, crystal form, and the high degree of acetylation (DA = 96.03%) of ChNps did not change during regeneration, suggesting that ChNps retained the complete molecular structure of chitin. The average particle size of ChNps–PTX was approximately 93.11 nm, which was larger than that of ChNps (84.06 nm) because of loading PTX (the drug loading was approximately 8.01%). TEM and CLSM were employed to confirm PTX existence in ChNps–PTX, and the nano-strip PTX (30.52 ± 6.78 nm in length and 16.02 ± 2.77 nm in width) was found for the first time. Loading of PTX resulted in ChNps–PTX presenting a new characteristic peak at 1734 cm^−1^ in FT-IR spectra, a new peak at 2θ = 5.3° in XRD pattern, and a new exothermic peak (252 °C) in DSC curve. While ChNps–PTX showed a lower crystallinity (19.86%) compared with that of ChNps (24.11%) and chitin (36.77%), utilizing the chitin carrier and mild regeneration method to load PTX were highly beneficial for pharmaceutical fields.

## 1. Introduction

Chitin is widely found in the exoskeletons of crustaceans and mollusks [1], and shrimp and crab shells are the primary commercially available source. It is a biocompatible and biodegradable linear polysaccharide composed of β- (1,4) -2-acetamido-2-deoxy-β-D-glucose monomer units [2]. Chitin is generally insoluble in common solvents, resulting in a much lower usable frequency compared to other natural polysaccharides. Nevertheless, the advantages such as stability, dense structure, excellent mechanical strength, good compatibility with hydrophobic drugs, and resistance to acids and alkali, all of which enable it to protect and load hydrophobic drugs or active substances, originate from its insolubility. Chitin nanoparticles (ChNps) are widely used in fields such as agriculture, functional food, medicine, and composite materials [3]. Additionally, ChNps display small particle size and superior surface properties, which may be conducive to their application in the field of drug loading.

The preparation methods of ChNps play a decisive role in their key properties such as particle size, morphology, and structural characteristics, which in turn affects the practical application of chitin. Ordinary preparation methods include mechanical treatment, acid hydrolysis, emulsification, and regeneration. Mechanical treatment consumes large amounts of physical energy [4]. Acid hydrolysis is prone to reduce the degree of acetylation (DA) of chitin and further to enhance the hydrophilicity of chitin [5]. The emulsifying process can accompany high energy consumption [6]. Regeneration is one of the most convenient methods for preparing ChNps. Its preparation principle lies in disrupting the dissolved state of chitin solution, thereby inducing the desolvation, aggregation, and phase separation of the chitin molecular units to spontaneously arrange into various forms such as nanoparticles [7]. Previous papers have reported that regenerating ChNps can be prepared by adjusting pH, or by adding anti-solvent to its original solvent like water and methanol [8,9,10]. The regeneration method can also facilitate the direct preparation of drug-loaded nanoparticles. For example, curcumin-loaded nanoparticles were obtained using methanol as the anti-solvent [11]. However, the regeneration method of adjusting pH often requires the use of high concentration acids and alkalis, which may cause a slight decrease in the DA of chitin [12]. On the other hand, nanoparticles prepared by conventional methods exhibited poor aggregative stability, which may be associated with factors such as pH, entropy, and microgravity [13,14,15]. The agglomeration could exert adverse impacts on practical applications [16]. The occurrence of aggregation in ChNps has been verified by the SEM results reported in previous literature [17]. The regeneration method of adding water drop-by-drop might be an ideal method for preparing ChNps, because it is more likely to retain the original hydrophobic structure of chitin and improve the aggregative stability.

Paclitaxel (PTX) is a hydrophobic drug with a high melting point and one of the most effective natural anti-cancer drugs as is known to all [18]. However, the direct use of PTX can lead to toxicity and low bioavailability [19]; thus, an appropriate drug loading is needed. The particle size and surface properties of carriers are crucial factors affecting drug bioavailability [20]. Drug-loaded nanoparticles can achieve transmembrane transport to enter the humoral circulation and reach target cells, thereby improving the bioavailability of the drugs, which was widely applied in small intestinal drug delivery [21,22,23]. Chitin with acetyl hydrophobic groups is suitable for loading PTX. Previous papers have reported PTX loaded into low DA chitin that is under 58.7% (chitosan), and the carriers were modified or cross-linked [24,25,26]. Not only does this cause chitin to lose its inherent excellent properties, but also there are potential health risks associated with the metabolism of the loaded drug. There has been no report related to the direct loading of PTX using chitin with a high DA so far. Therefore, it is urgently needed to develop a simple method for preparing small and uniform high DA ChNps loaded with PTX.

In this work, a new water-dripping regeneration method was established for preparing ChNps and ChNps–PTX. The mild preparation method can completely retain the molecular structure of chitin and ensure well-dispersed nanoparticles. Moreover, it achieved the effective loading of paclitaxel nanoparticles into chitin with a high DA for the first time. The microstructure of ChNps and ChNps–PTX was systematically characterized using Scanning Electron Microscopy (SEM), Transmission Electron Microscopy (TEM), Fourier Transform Infrared (FT-IR), Differential Scanning Calorimetry (DSC), X-ray Diffraction (XRD), and Confocal Laser Scanning Microscopy (CLSM). The research is expected to expand the application of chitin in the functional food and pharmaceutical fields and promote the development of PTX in drug delivery.

## 2. Materials and Methods

### 2.1. Materials

Chitin (with a DA of 96%) was purchased from Zhejiang Jinke Biochemical Co. (Yuhuan, China). Paclitaxel (PTX) was purchased from Xi’an Jiahe Biotechnology Co. (Xi’an, China). Methanol and Calcium chloride dihydrate were obtained from Sinopharm Chemical Reagent Co. (Shanghai, China). Deionized water (with a conductivity of approximately 0.32 µS/cm) was used throughout the experiment.

### 2.2. Preparation of Chitin Solution

Following the method described by Hiroshi Tamura et al. [27] with slight modifications, 82 g calcium chloride was mixed with 100 mL methanol. The mixture was stirred at 30 °C for several hours until an insoluble precipitate remained. After centrifugation, 0.4 g chitin was added to the supernatant (the initial chitin solution concentration was around 4 mg/mL). The mixture was stirred at 30 °C for 24 h, after which the precipitate was removed by centrifugation to obtain the chitin solution.

### 2.3. Preparation of ChNps

ChNps were prepared from the chitin solution using the regeneration method. The chitin was dissolved in a saturated methanol-calcium chloride solution with water used as the anti-solvent and added in a dropwise manner to induce the precipitation of chitin. The method was carried out based on the work of Zhang et al. and Sabitha M et al. [9,11] with improvements. In total, 10 mL deionized water was added dropwise into 10 mL chitin solution at 4500 rpm. The mass ratio of water and chitin solution was 1:1. The mixture was stirred at 1000 rpm for 30 min to make the solution turbid. The precipitate was collected by centrifugation and cleansed with water to remove methanol and calcium chloride until the conductivity approached that of the deionized water tested. The precipitate was then freeze-dried to obtain ChNps.

### 2.4. Preparation of ChNps–PTX

In total, 0.01 g PTX was dissolved in 10 mL chitin solution to form a Chitin–PTX co-solution. Then, 10 mL deionized water was added dropwise into the co-solution at 4500 rpm. The mass ratio of water and Chitin–PTX co-solution was 1:1. Then the mixture was stirred at 1000 rpm for 30 min, during which the solution gradually changed from clear to turbid. The precipitate was collected by centrifugation and rinsed repeatedly with deionized water to remove residual methanol and calcium chloride. Subsequently, ethanol was added to wash off unbounded PTX remaining on the surface of the sample. Finally, deionized water was used again to rinse away ethanol, and the conductivity of the rinse solution was measured to confirm thorough cleaning. The cleaned precipitate was freeze-dried to obtain ChNps–PTX.

### 2.5. Determination of DA for Chitin and ChNps

The DA of chitin was determined via Proton Nuclear Magnetic Resonance Spectroscopy (^1^H-NMR spectra). The sample was freeze-dried repeatedly using 99% D_2_O (pH = 4) which maximized the reduction in water interference through deuterium atoms [28]. The sample was analyzed using a Bruker ARX 300 NMR spectrometer. The DA of chitin was calculated according to Equation (1) [29].

*DA* = (1/3 × *I_CH_*_3_)/(1/6 × *I*_(*H*2–*H*6)_) (1)

where *I_CH_*_3_ is the integral area ratio of -CH_3_ signal (located at 1.94–2.46 ppm), and *I*_(*H2*–*H6*)_ refer to the total integral area of H2–H6 protons (H2, H3, H4, H5, H6) (3.17–4.34 ppm).

### 2.6. Determination of Drug Loading (DL)

The content of PTX was determined by high-performance liquid chromatography (HPLC) utilizing a C18 reversed-phase chromatographic column (Kromasil 100-5-C18, 4.6 mm × 250 mm, Nouryon Specialty Chemicals). (Amsterdam, The Netherlands). The column temperature was maintained at approximately 25 °C, and the ultraviolet detection wavelength for PTX was 227 nm. In total, 10 μL solution was injected into the HPLC instrument. The mobile phase consisted of methanol, deionized water, and acetonitrile with a volume ratio of 52.5:25:22.5, and the flow rate was set at 0.5 mL/min [30].

In total, 0.005 g PTX (10 μg/mL) was dissolved in 50 mL methanol to obtain the PTX solution. Subsequently, 5 mL of the PTX solution was taken and made up to 10 mL with methanol. Using this method, the solution was gradually diluted until standard solutions with PTX concentrations ranging from 10 to 0.625 μg/mL were obtained. The peak areas of PTX were determined by HPLC, which were used to construct the standard curve with peak area versus concentration.

The DL of ChNps–PTX could be calculated using the following method. After regeneration of ChNps–PTX, 1 mL of the mixed solution was taken and diluted with methanol to a constant volume of 100 mL. The content of free paclitaxel (PTX) was determined by high-performance liquid chromatography (HPLC), so as to calculate the mass of paclitaxel loaded in ChNps–PTX. The equation for DL referred to Equation (2).

*DL* = (*m*_1_ − *m*_2_)/*m*_3_ (2)

where *m*_1_ is the addition weight of PTX in the ChNps–PTX preparation process, *m*_2_ refers to the remaining weight of PTX after preparation of ChNps–PTX, and *m*_3_ is the weight of ChNps–PTX.

### 2.7. Characterization

SEM images were obtained using a Phenom Pro SEM system (Phenom Scientific Instrument Co.) (Eindhoven, The Netherlands). The sample surface was sputter-coated with Nano gold, and observations were performed at an accelerating voltage of 10 kV to determine the apparent morphology and particle size. A Tecnai JEM-F200 microscope (JEOL Ltd., Tokyo, Japan) was used for TEM analysis. The particle size was measured using the particle size analysis tool (Nano Measure) (Beihang University, V1.2), and 20 sets of data were selected for the calculation of the average value and standard deviation. ChNps–PTX was ultrasonically dispersed in ethanol, and the suspension was sampled onto a hole-free carbon film, followed by drying prior to detection. CLSM (Model TCS SP8 X, Leica Microsystems) (Wetzlar, Germany) was used to obtain fluorescence images of sample component distribution. ChNps–PTX was pre-stained with Nile Blue and Nile Red, and excess dye on the sample surface was rinsed off with deionized water. After uniform ultrasonic dispersion, the samples were observed under the excitation wavelengths of the two dyes (488 and 633 nm), respectively. Fourier FT-IR spectroscopy was conducted using a Thermo Scientific NICOLET iS10 spectrometer (Thermo Fisher Scientific, Massachusetts, USA) in the range of 400–4000 cm^−1^ with 36 scans. Samples were prepared at a ratio of sample to potassium bromide (KBr) = 1:100, ground, pressed into pellets, and then analyzed. XRD measurements were carried out using a Bruker D8 Advance diffractometer (Bruker Corporation, Massachusetts, USA) with a scanning range of 0° to 60° (2θ), operating at a tube voltage of 40 kV and a tube current of 30 mA. DSC was performed using a 214 Polyma instrument (NETZSCH-Gerätebau GmbH, Selb, Germany) under a nitrogen atmosphere (50 mL/min). The heating rate was 10 °C/min, with a temperature range of 30–280 °C.

## 3. Results and Discussion

For the anti-solvent regeneration method, the solute molecules were induced to precipitation when a kind of anti-solvent was added to its original solution to disrupt the dissolve state, and the nucleation and nucleus aggregation occurred simultaneously [31]. Adding a large amount of anti-solvent directly tends to cause agglomeration of the product [32], whereas adding the anti-solvent via a dripping method was conducive to promoting the slow occurrence of the nucleation process and was beneficial for the formation of small-sized nanoparticles. Moreover, a shear force generated by vigorous stirring was beneficial to the dispersion of nanoparticles [8]. Thus, a water-dripping regeneration method was innovatively used to prepare the small and uniform ChNps and ChNps–PTX and avoid the destruction of the original hydrophobic structure of chitin. ChNps–PTX was prepared by the regeneration of PTX and chitin after they formed a co-solution, where the preparation of nanoparticles and the drug-loading process occur simultaneously. The DL was approximately 8.01%.

### 3.1. DA Analysis of Chitin and ChNps

**Figure 1:**
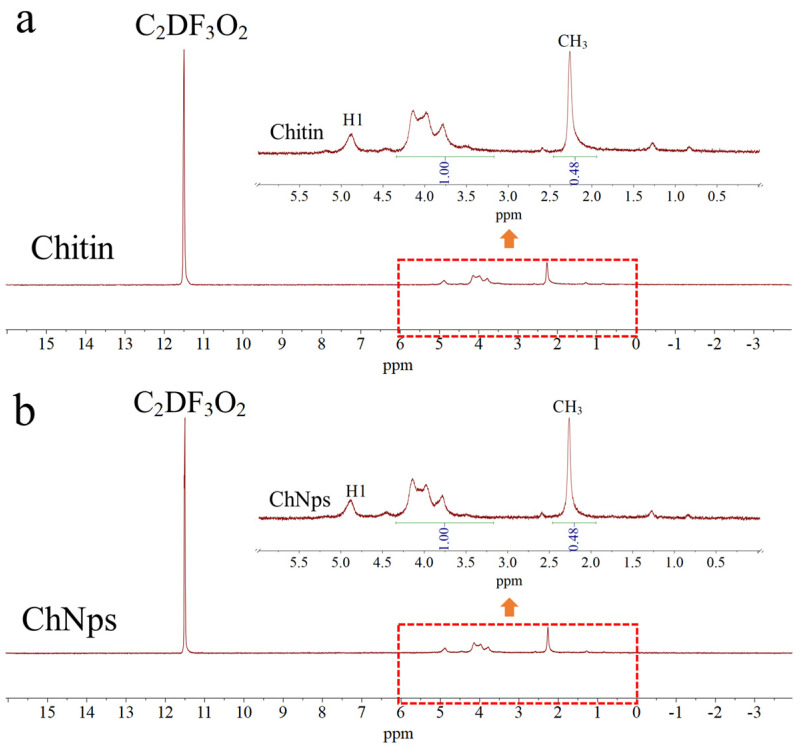
^1^H-NMR spectra and of chitin (**a**) and ChNps (**b**).

Losing the hydrophobic groups of chitin has an adverse effect on the loading of hydrophobic drugs, so the acetyl groups of ChNps should be retained during the production process. ^1^H-NMR was a highly accurate method for determining the DA of chitin. The CF_3_COOD solvent enabled the detection of chitin over a wide DA range, as it can dissolve chitin with high DA [33]. Figure 1 presented the ^1^H-NMR spectra of raw chitin and ChNps. The repeat unit of chitin contained key proton environments: H1 (the anomeric proton on the glucose ring) exhibited a chemical shift in the range of 4.5–5.5 ppm. The methyl protons of the acetyl group (CH_3_CO-) (linked to the carbonyl and nitrogen atoms) presented a chemical shift between 1.94 and 2.46 ppm. H2–H6 (other protons on the glucose ring) showed chemical shifts in the range of 3.17–4.34 ppm. The spectra of the two samples were basically consistent, indicating that the molecular backbone structure of chitin was unchanged after regeneration. The slight differences in peak shape and intensity between the two spectra might be attributed to factors such as the particle size and surface properties of ChNps. The integral areas and the calculated DA values of raw chitin and ChNps were summarized in Table 1. The results displayed that the DA values of raw chitin and ChNps were both 96.03%, confirming that the dissolution and subsequent regeneration process did not reduce the DA of chitin. It has been reported in a previous paper that chitin dissolved in phosphoric acid led to a decrease in DA from 91% to 77%~87% [34]. ChNps loaded with curcumin showed a DA of 72.4% [11]. And the DA of two kinds of chitosan determined by ^1^H-NMR spectrum was 28% and 3%, respectively [29]. The dissolution of chitin in saturated calcium chloride-methanol solution and the preparation of ChNps via water-dripping regeneration method were both physical processes, thus remaining at a high DA equal to the raw chitin. The ChNps still retained a high content of hydrophobic groups, preserving the structural advantages of chitin and facilitating its combination with the hydrophobic drug PTX.

### 3.2. Morphological Analysis

**Figure 2:**
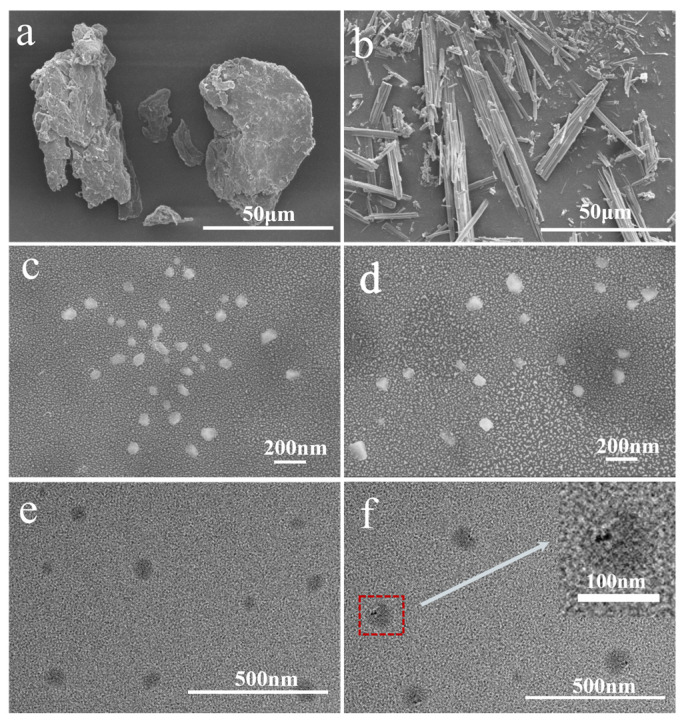
SEM images of chitin (**a**), PTX (**b**), ChNps (**c**), and ChNps–PTX (**d**); TEM images of ChNps (**e**) and ChNps–PTX (**f**).

**Figure 3:**
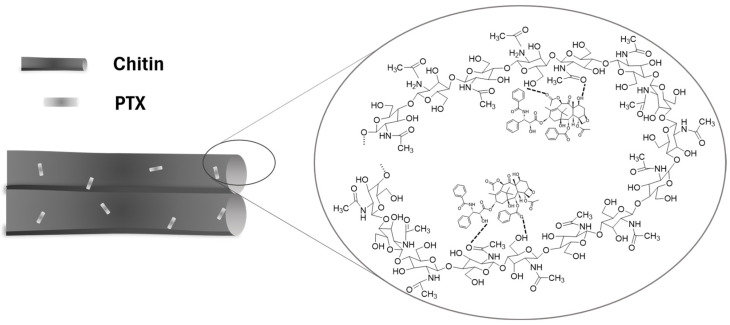
The loading schematic diagram of ChNps–PTX.

The SEM was used to observe the apparent morphology of the samples, while the TEM was applied to directly observe the PTX loading status. Figure 2 presented the SEM and TEM results of chitin, PTX, ChNps, and ChNps–PTX. SEM images showed that chitin (Figure 2a) exhibited an irregular blocky structure, and PTX (Figure 2b) presented a long crystalline strip morphology. The ChNps (Figure 2c) and ChNps–PTX (Figure 2d) prepared by the water-dripping regeneration method showed relatively uniform nano-quasi-spherical particles and excellent dispersibility. The morphology of ChNps (Figure 2e) and ChNps–PTX (Figure 2f) observed by TEM was consistent with that of SEM. The particle sizes of ChNps, ChNps–PTX, and the loaded PTX were obtained using the particle size measurement tool (Nano Measure), which has been previously adopted in existing studies [35]. The average particle size of ChNps–PTX (approximately 93.11 nm) was larger than that of ChNps (approximately 84.06 nm) due to the loading of PTX. In comparison, the ChNps prepared in this work exhibited a smaller particle size and better dispersibility than the ChNps with a particle size of approximately 150 nm prepared by the ionic gelation method reported in previous studies [17,36]. It has been reported that well-dispersed ChNps with a particle size of 70~80 nm were regenerated via the dropwise addition of methanol [11]. These results indicated that water-dripping and vigorous stirring were efficient approaches to promote the reduction in particle size and achieve uniform dispersion. The loading of PTX into ChNps was directly observed for the first time, and the PTX exhibited nano-strips 30.52 ± 6.78 nm in length and 16.02 ± 2.77 nm in width, as seen in Figure 2f. The loading schematic diagram of ChNps–PTX was shown in Figure 3. During the precipitation process, chitin chains and PTX may bind to each other due to the presence of interactions such as hydrogen bonds and van der Waals forces.

### 3.3. CLSM Analysis

**Figure 4:**
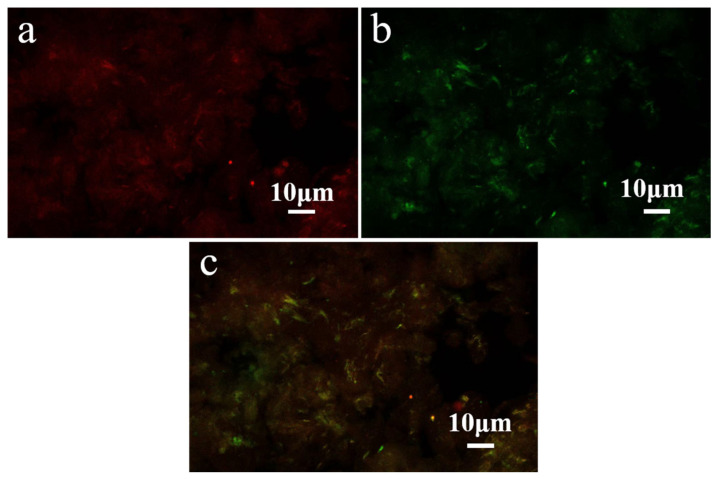
CLSM images of ChNps–PTX under irradiation with 633 nm (**a**) and 488 nm (**b**) wavelengths, and superimposed laser channels (**c**).

CLSM was used to characterize the loading system of ChNps–PTX, and the results are presented in Figure 4. ChNps–PTX stained with Nile Red exhibited red dots or filaments under excitation at 633 nm, while ChNps–PTX stained with Nile Blue showed green dots or filaments under excitation at 488 nm. As can be seen in Figure 4c, the merged laser channel image of the two signals displayed an orange-yellow color, which directly confirmed the successful loading of PTX in ChNps. This result was consistent with the findings of Xuechen Zhuang et al. [30], who successfully achieved loading PTX into debranched corn starch.

### 3.4. Structural Characterization

#### 3.4.1. FT-IR Analysis

**Figure 5:**
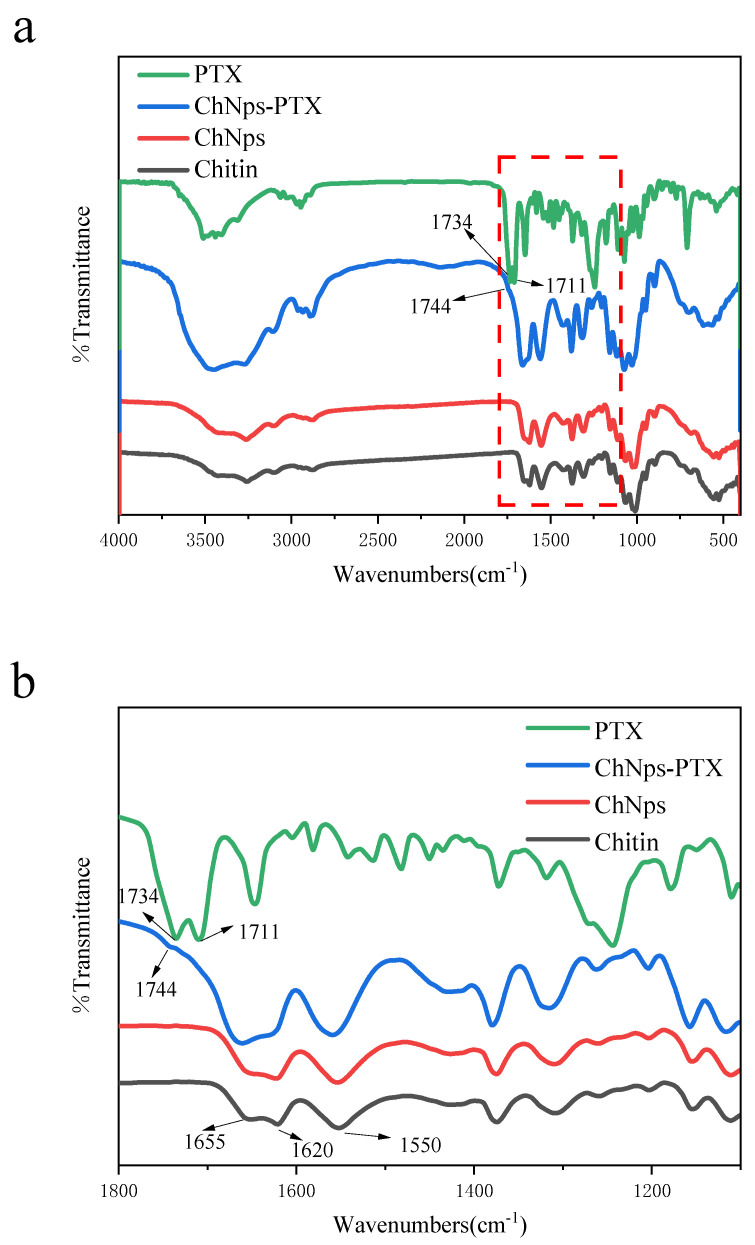
FT-IR spectra (**a**) and partially enlarged FT-IR spectra from 1800 to 1100 cm^−1^ (**b**) of chitn, ChNps, ChNps–PTX, and PTX.

Figure 5 displays the FT-IR spectra of chitin, ChNps, ChNps–PTX, and PTX. The peak bands in the 3430–3100 cm^−1^ region represented the stretching of -OH and -NH groups, which were characteristic of intermolecular hydrogen bond networks [37]. The vibration regions of the chitin and ChNps curves were basically consistent, indicating that the chemical structure of ChNps prepared by the water-dripping regeneration method was unchanged. Moreover, the amide I band (C=O) observed around 1655 and 1620 cm^−1^ and the amide II band (N-H) observed at 1550 cm^−1^ of the ChNps spectrum were similar to that of the raw chitin, proving that the crystal form of α-chitin has not changed after regeneration [38]. This result was consistent with the previous report [39], attributed to the unchanged functional groups of chitin during the regeneration process. For PTX, the peak at 3456 cm^−1^ was the specific peak of PTX’s hydroxyl groups (O-H), and the peaks at 2850 cm^−1^ and 2917 cm^−1^ correspond to the C-H stretching vibration [8]. The peak shown at 1646 cm^−1^ was N-H stretching vibration, and 1243 cm^−1^ was C-N stretching vibration. The peak at 709 cm^−1^ was C-C stretching vibration. The peaks at 1734 and 1711 cm^−1^ of PTX represented the carbonyl (C=O) stretching vibration [18]. In Figure 5b, ChNps–PTX exhibited a new peak at 1744 cm^−1^, which indicated the presence of C=O signal of PTX. This result confirmed the successful loading of PTX into ChNps.

#### 3.4.2. XRD Analysis

**Figure 6:**
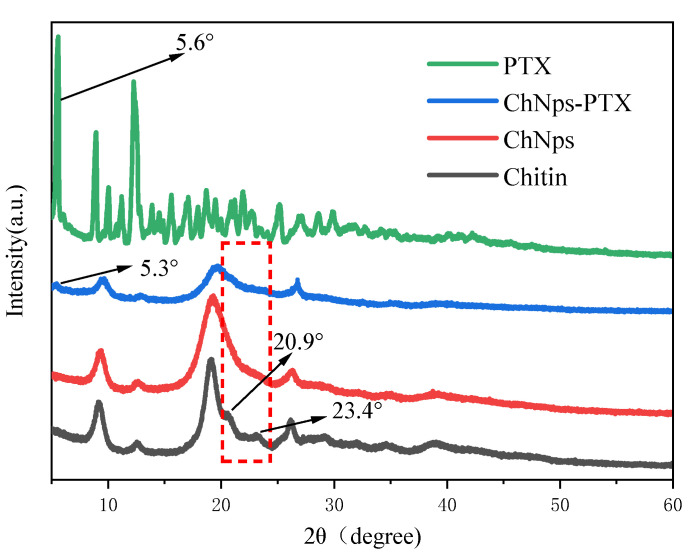
XRD patterns of chitin, ChNps, ChNps–PTX, and PTX.

Figure 6 presented the XRD patterns of chitin, ChNPs, ChNps–PTX, and PTX. For the chitin and ChNps curves, distinct diffraction peaks appeared at 2θ = 9.3°, 12.6°, 19.5°, 22.8°, 23.3°, and 26.5°, representing that their crystalline forms were both α-chitin [40]. This was consistent with the FT-IR results (Section 3.4.1). Compared with chitin, the diffraction peaks of ChNPs and ChNps–PTX located at 2θ = 20.9° and 23.4° disappeared, and the degree of crystallinity of chitin, ChNps, and ChNps–PTX was 36.77%, 24.11%, and 19.86%, respectively. These results indicated a reduction in crystallinity of ChNPs and ChNps–PTX, which was consistent with previous papers related to the regenerated chitin [38,41]. The decreased crystallinity of ChNps was attributed to the rapid arrangement of chitin during the regeneration process, which led to the inadequate development of crystallization and chitin chains [42]. Furthermore, ChNps–PTX exhibited even lower crystallinity, which may be related to the disruption of the original crystalline structure by the interaction between chitin and PTX. PTX exhibited obvious characteristic diffraction peaks at multiple 2θ values, including 5.56°, 8.8°, and 12.4°. Compared with chitin and ChNps, ChNps–PTX showed a new diffraction peak at 2θ = 5.3°, further proving the successful loading of PTX into ChNps.

#### 3.4.3. DSC Analysis

**Figure 7:**
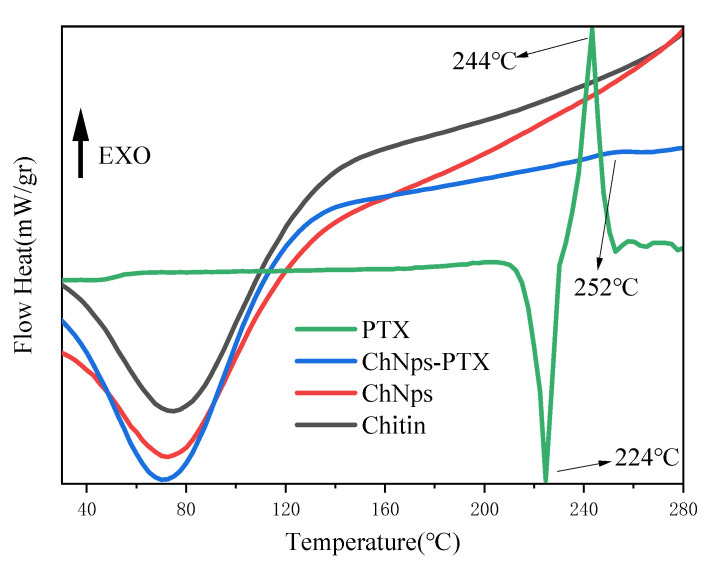
DSC thermograms of chitin, ChNps, ChNps–PTX, and PTX.

Chitin possessed good thermal stability due to its rigid hydrogen bond structure and acetyl groups. Figure 7 displayed the DSC thermograms of chitin, ChNps, ChNps–PTX, and PTX. Since polysaccharides tend to bind with water [43], the first endothermic peaks of chitin, ChNps, and ChNps–PTX appearing below 100 °C were attributed to the evaporation of adsorbed water [44], which was in agreement with the conclusions reported in a previous paper [45]. As the temperature continued to rise, thermal decomposition and release of volatile products occurred. For PTX, the endothermic peak at 224 °C and exothermic peak at 244 °C corresponded to its melting and decomposition processes, respectively [46]. A new exothermic peak of ChNps–PTX emerging at 252 °C compared with chitin and ChNps may be caused by the interaction between chitin and PTX, which also confirmed the successful loading of PTX into ChNps.

## 4. Conclusions

In this research, PTX and chitin were dissolved in the same solvent to form a co-solution, and the water-dripping regeneration method was used to prepare ChNps and ChNps–PTX with a relatively uniform particle size of approximately 84.06 and 93.11 nm, respectively. This study realized the loading of PTX into ChNps in the form of nano-strips (30.52 ± 6.78 nm in length and 16.02 ± 2.77 nm in width) for the first time, and the DL was approximately 8.01%. A series of characterizations demonstrated that the functional groups, DA, and crystal form of chitin were unchanged after regeneration, which was different from previous papers. ^1^H NMR spectra demonstrated that chitin and ChNps both showed high DA of 96.03%, indicating that the ChNps retained the acetyl groups. TEM and CLSM directly confirmed that PTX was loaded into ChNps rather than existing in a free state. The results of FT-IR and XRD displayed that the water-dripping regeneration method did not damage the molecular structure of chitin, and ChNps still had α-chitin structure. For ChNps–PTX, the new peak of 1734 cm^−1^ in the FT-IR spectrum, 2θ = 5.3° in the XRD pattern, and a new exothermic peak of 252 °C in the DSC curve confirmed the successful loading of PTX into ChNps. Thus, ChNps ensure stable PTX loading through simple methods, which will provide a feasible solution for the improvement of bioavailability of PTX. It is also beneficial for the clinical application of PTX. We believe that the ChNps and ChNps–PTX prepared in this work will provide a reference for the research of chitin carriers and broaden the applications of chitin in the food and pharmaceutical fields. The water-dripping regeneration method can further expand the research and development of composite carriers for loading fat-soluble drugs and construct a multifunctional drug delivery system.

## Figures and Tables

**Table 1:** Integral areas and DA of chitin and ChNps.

Sample	Integral Area of -CH_3_ (1.94–2.64 ppm)	Integral Area of H2–H6 (3.17–4.34 ppm)	DA, %
**Chitin**	0.48	1.00	96.03
**ChNps**	0.48	1.00	96.03

## Data Availability

The original contributions presented in this study are included in the article. Further inquiries can be directed to the corresponding authors.

## Abbreviations

The following abbreviations are used in this manuscript:

ChNps	Chitin nanoparticles
PTX	Paclitaxel
ChNps–PTX	Chitin nanoparticles–paclitaxel
DA	degree of acetylation
DL	drug loading
FT-IR	Fourier Transform Infrared
DSC	Differential Scanning Calorimetry
XRD	X-ray Diffraction
CLSM	Confocal Laser Scanning Microscopy
TEM	Transmission Electron Microscopy
SEM	Scanning Electron Microscopy
^1^H-NMR	Proton Nuclear Magnetic Resonance Spectroscopy
HPLC	high-performance liquid chromatography
